# Associations of lifestyle behaviors with mental health in a nationwide cross-sectional survey of 152,860 Brazilian students

**DOI:** 10.1590/1984-0462/2025/43/2024080

**Published:** 2024-10-28

**Authors:** Eduardo Rossato de Victo, Gerson Ferrari, Clemens Drenowatz, Dirceu Solé

**Affiliations:** a Universidade Federal de São Paulo, Escola Paulista de Medicina, São Paulo, SP, Brazil; b Universidad de Santiago de Chile, Escuela de Ciencias de la Actividad Física, el Deporte y la Salud, Santiago, Chile.; c Universidad Autónoma de Chile, Facultad de Ciencias de la Salud, Providencia, Chile.; d University of Education Upper Austria, Division of Sport, Physical Activity and Health, Linz, Austria.

**Keywords:** Lifestyle, Mental health, Adolescent, Estilo de vida, Saúde mental, Adolescente

## Abstract

**Objective:**

To evaluate the association between modifiable lifestyle behaviors and mental health indicators in Brazilian adolescents.

**Methods:**

This cross-sectional study used data from the 2019 National Survey of School Health including 152,860 students. The lifestyle behaviors and mental health indicators were obtained from a self-reported questionnaire. Logistic regression analyses were used to examine the association between lifestyle behaviors and mental health indicators.

**Results:**

Infrequent healthy eating (OR 1.22; 95%CI 1.14–1.31), being inactive (OR 1.82; 95%CI 1.65–2.01), smoking (OR 1.24; 95%CI 1.10–1.40), and irregular school attendance (OR 1.31; 95%CI 1.22–1.40) were associated with not having close friends. Additionally, not having close friends was also associated with unhealthy eating (OR 0.86; 95%CI 0.81–0.92) and alcohol consumption (OR 0.81; 95%CI 0.75–0.87). Infrequent healthy eating (OR 1.29; 95%CI 1.24–1.33), frequent unhealthy eating (OR 1.39; 95%CI 1.35–1.43), being inactive (OR 1.12; 95%CI 1.07–1.18), excessive screen time and sitting (OR 1.10; 95%CI 1.07–1.14 and OR 1.68; 95%CI 1.63–1.73, respectively), smoking (OR 1.26; 95%CI 1.19–1.34), alcohol (OR 1.33; 95%CI 1.28–1.37), drugs (OR 1.13; 95%CI 1.05–1.22), and irregular school attendance (OR 1.53; 95%CI 1.48–1.59) were associated with worse self-rated mental health.

**Conclusions:**

Various lifestyle behaviors are associated with different indicators of mental health.

## RESUMO

**Objetivo:**

Avaliar a associação entre comportamentos modificáveis do estilo de vida e indicadores de saúde mental em adolescentes brasileiros.

**Métodos:**

Este estudo transversal utilizou dados da Pesquisa Nacional de Saúde do Escolar de 2019, incluindo 152.860 alunos. Comportamentos de estilo de vida e indicadores de saúde mental foram obtidos por meio de um questionário autoaplicável. Análises de regressão logística foram utilizadas para examinar a associação entre comportamentos de estilo de vida e indicadores de saúde mental.

**Resultados:**

Alimentação saudável infrequente (OR 1,22; IC95% 1,14–1,31), ser inativo (OR 1,82; IC95% 1,65–2,01), tabagismo (OR 1,24; IC95% 1,10–1,40) e frequência escolar inadequada (OR 1,31; IC95% 1,22–1,40) foram associados a não ter amigos próximos. Além disso, não ter amigos próximos também foi associado com alimentação não saudável (OR 0,86; IC95% 0,81–0,92) e consumo de álcool (OR 0,81; IC95% 0,75–0,87). Alimentação saudável infrequente (OR 1,29; IC95% 1,24–1,33), alimentação não saudável frequente (OR 1,39; IC95% 1,35–1,43), ser inativo (OR 1,12; IC95% 1,07–1,18), tempo excessivo em tela e sentado (OR 1,10; IC95% 1,07–1,14 e OR 1,68; IC95% 1,63–1,73, respectivamente), tabagismo (OR 1,26; IC95% 1,19–1,34), álcool (OR 1,33; IC95% 1,28–1,37), drogas (OR 1,13; IC95% 1,05–1,22) e frequência escolar irregular (OR 1,53; IC95% 1,48–1,59) foram associados a uma pior autopercepção de saúde mental.

**Conclusões:**

Vários comportamentos de estilo de vida foram associados a diferentes indicadores de saúde mental.

## INTRODUCTION

Adolescence is a timespan during human development, making it an important period for establishing the foundations for good health later in life.^1^ During this period, adolescents undergo physical, cognitive, and psychosocial development that leads them to establish standards of behaviors that can protect them or put their physical and mental health at risk.^1^ According to the World Health Organization (WHO), adolescents with mental health problems are more vulnerable to social exclusion and discrimination, presenting educational difficulties and risk behaviors, in addition to worse physical health.^2^

Worldwide, it is estimated that 10 to 20% of adolescents suffer from some mental health disorder.^3^ In Brazil, data from the United Nations Children’s Fund show that one in every six adolescents, between 10 and 19 years of age, displays an indicator of mental health disorder with self-mutilation, depression, and suicide being the most common ones.^4^ Furthermore, low- and middle-income countries, such as Brazil, present difficulties in reporting systems for mental health disorders, generating possible underreporting of cases, especially those related to suicidal behaviors and ideation.^5^

Several indicators have been used to assess mental health, including the number of close friends, as it portrays the possible social isolation of the adolescent. According to a study that compiled data from 90 countries around the world, the number of close friends was inversely associated with suicidal thoughts and suicide attempts.^6^ Another important indicator is self-assessment of mental health, as it is based on questions relating to feelings of sadness, irritation, nervousness, lack of concern about the perception of others, and that life is not worth living.^7,8^

Mental health is shaped by genetic, environmental, social, and personal factors, and researchers have investigated those that may be associated with better or worse mental health.^9,10^ With changes in adolescents’ lifestyles throughout the 21st century, there is also a need to understand and include possible new risk factors and behaviors associated with mental health.^10^

Since 2001, the WHO has recommended research on biological and psychosocial aspects of mental health in order to better understand their interactions, in addition to reinforcing the importance of research using national data.^11^ Previously published studies also encouraged new research to enhance the understanding of the relationship between various behaviors and the mental health of Brazilian adolescents.^12^ Therefore, the objective of this study was to evaluate the association between modifiable lifestyle behaviors and mental health indicators in Brazilian adolescents.

## METHOD

The present study is a secondary, cross-sectional analysis of existing data from the National Survey of School Health (PeNSE), from the year 2019.^7,13^ PeNSE is a survey carried out by the Brazilian Institute of Geography and Statistics (IBGE) on Brazilian adolescents, whose objective is to investigate risk and protective factors for schoolchildren’s health. Its results aim to guide the development of public policies to improve the health of schoolchildren by identifying the main issues for the country.^13^ The data is publicly accessible and available for use. All participants provided Informed Consent and the research was approved by the responsible institutions and secretaries. The PeNSE 2019 was approved by the National Research Ethics Commission, opinion no. 3,249,268, dated 04/08/2019.

The first edition of PeNSE was conducted in 2009, with subsequent evaluations in 2012, 2015, and 2019. The 2019 PeNSE introduced substantial modifications, including a more comprehensive questionnaire encompassing new topics such as mental health, in addition to the traditional topics of nutrition, physical activity, and substance use. The sample size was expanded to comprise adolescents aged 13 to 17 years from all regions of Brazil, covering both urban and rural areas, and encompassing both public and private schools. The research used a two-stage sampling technique, with the first stage being the selection of schools and the second being the selection of classes. In total, adolescents from all regions and states of Brazil, including Brasília, participated in this study. The initial plan included 1,288 municipalities and 4,361 schools across the country. The study’s initial sample was 165,838 adolescents. Those who did not present valid answers to one or more of the variables analyzed were eliminated. Exceptions were missing data in the variables: smoking, alcohol and drug consumption. This exception was justified by the magnitude of sample loss that occurred since only 17.9% provided information on smoking, 53.1% for alcohol consumption, and 10.6% for drug consumption. After considering these exclusion criteria, the final sample was 152,860 adolescents.

Data were collected using a self-administered questionnaire structured according to thematic blocks with smartphones being used for its completion. Questions were in multiple-choice format and the participants were given the possibility of not answering any question or the entire questionnaire. There was also a help icon when filling out the questionnaire that guided the teenagers. Additional details about the questionnaire, questions used and possible answers, research reports and microdata can be found on the website and in the PeNSE document itself^7^ (https://www.ibge.gov.br/estatisticas/sociais/educacao/2051-pesquisa-nacional-de-saude-do-escolar/9134-pesquisa-nacional-de-saude-do-escolar.html).

The usual consumption of healthy food was determined by the intake of fresh or minimally processed foods according to the average weekly ingestion of beans, fruits, vegetables, or greens. Average consumption of less than five times/week was classified as “infrequent”.^7,14^ The habitual consumption of unhealthy food was determined by the intake of sweet treats. Consumption on five or more days during the last seven days was classified as “frequent”.

Accumulated physical activity (PA) was obtained according to the sum of physical activities carried out in the week prior to the survey by the time spent on: 1) Commuting to and from school on foot or by bicycle; 2) Participation in physical education classes; and 3) Extracurricular activities. The total time of the last seven days was classified as “active” (300 or more minutes), “insufficiently active” (1 to 299 minutes), and “inactive” (zero).^7,14^

Sedentary behavior was measured by two situations: total screen time and sitting time. Total screen time of more than two hours/day was classified as “inadequate” and sitting time was classified as “inadequate” when it was more than three hours/day.^7^

Movement recommendations were: being “active” (300 or more minutes), “adequate” screen time (two or fewer hours/day), and “adequate” sitting time (three or fewer hours/day).^7,14^

Information on smoking, alcohol consumption, and drug use (marijuana, cocaine, among others) was obtained by reporting their respective consumption in the last 30 days prior to the survey, being classified as “yes” when consumed at least once during the period.^7,15^

School attendance was evaluated according to the number of days the student missed classes or school without permission from parents/guardians in the 30 days prior to the survey. It was classified as “inadequate” when the student reported at least one unexcused absence from school during the period.^7,15^

Mental health was analyzed using two indicators. One of them was the number of close friends and when “none” was mentioned, it was classified as a risk factor.^7^

Another indicator was self-assessed mental health, based on five questions addressing:

1. Feeling of concern;
2. Feeling of sadness;
3. Feeling that no one cares about them;
4. Feeling of irritation, nervousness, or bad mood; and
5. Feeling that life is not worth living. If the answer was reported as “most of the time” or “always” to at least four of the five questions, the self-assessment of mental health was classified as “negative”.

All questions referred to the last 30 days prior to the survey.^7,8^

Lifestyle factors were considered for analysis as independent variables and mental health indicators as dependent variables. Sex, age, and region were included as covariates in the analyses.

The comparison between the groups was conducted using the chi-square test. Logistic regression analyses were used to determine odds ratios (OR) and confidence intervals (95%CI). The values were expressed for the unadjusted and adjusted regression models. A possible existence of multicollinearity between the independent variables and the adjustment variables was evaluated using VIF (variance inflation factor) <10 and tolerance >0.8. The significance level was set at p<0.001. IBM Statistical Package for Social Sciences (SPSS) program for Windows, version 22 (IBM, 2013) was used for the analyses.

## RESULTS

The sample of the present study consisted of 152,860 adolescents (51.1% female). In total, 4,904 (3.2%) did not have close friends and 24,915 (16.3%) had a negative self-assessment of their mental health (Tables 1 and 2).

**Table 1:** Description of demographic characteristics and eating modifiable behaviors.

Total=152,860		Close friends			Mental health self-assessment		
		Risk (no friends) n (%) 4904 (3.2)	Healthy (with friends) n (%) 147956 (96.8)	p-value	Negative n (%) 24915 (16.3)	Positive n (%) 127945 (83.7)	p-value
Sex							
Male	74721 (48.9)	2595 (3.5)	72126 (96.5)	<0.001	6011 (8.0)	68710 (92.0)	<0.001
Female	78139 (51.1)	2309 (3.0)	75830 (97.0)		18904 (24.2)	59235 (75.8)	
Age							
<13 years old	24711 (16.2)	455 (1.8)	24256 (98.2)	<0.001	2600 (10.5)	22111 (89.5)	<0.001
13 to 15 years old	79351 (51.9)	2316 (2.9)	77035 (97.1)		13249 (16.7)	66102 (83.3)	
16 or 17 years old	40898 (26.8)	1625 (4.0)	39273 (96.0)		7850 (19.2)	33048 (80.8)	
≤18 years old	7900 (5.1)	508 (6.4)	7392 (93.6)		1216 (15.4)	6684 (84.6)	
Geographic region							
North	34084 (22.2)	1293 (3.8)	32791 (96.2)	<0.001	5912 (17.3)	28172 (82.7)	<0.001
Northeast	53470 (35.0)	1681 (3.1)	51789 (96.9)		8315 (15.6)	45155 (84.4)	
Southeast	27291 (17.9)	804 (2.9)	26487 (97.1)		4451 (16.3)	22840 (83.7)	
South	16593 (10.9)	415 (2.5)	16178 (97.5)		2531 (15.3)	14062 (84.7)	
Midwest	21422 (14.0)	711 (3.3)	20711 (96.7)		3706 (17.3)	17716 (82.7)	
Healthy eating							
Infrequent	116423 (76.2)	3925 (3.4)	112498 (96.6)	<0.001	20102 (17.3)	96321 (82.7)	<0.001
Frequent	36437 (23.8)	979 (2.7)	35458 (97.3)		4813 (13.2)	31624 (86.8)	
Unhealthy eating							
Frequent	48856 (32.0)	1395 (2.9)	47461 (97.1)	<0.001	10218 (20.9)	38638 (79.1)	<0.001
Infrequent	104004 (68.0)	3509 (3.4)	100495 (96.6)		14697 (14.1)	89307 (85.9)	

p-value of the chi-square test.

**Table 2:** Description of modifiable behaviors and mental health indicators of students.

Total=152,860		Close friends			Mental health self-assessment		
		Risk (no friends) n (%) 4904 (3.2)	Healthy (with friends) n (%) 147956 (96.8)	p-value	Negative n (%) 24915 (16.3)	Positive n (%) 127945 (83.7)	p-value
Physical activity level							
Inactive	14278 (9.3)	705 (4.9)	13573 (95.1)	<0.001	3251 (22.8)	11027 (77.2)	<0.001
Insufficiently active	95287 (62.4)	3018 (3.2)	92269 (96.8)		15659 (16.4)	79628 (83.6)	
Active	43295 (28.3)	1181 (2.7)	42114 (97.3)		6005 (13.9)	37290 (86.1)	
Screen time							
Excess	51918 (34.0)	1523 (2.9)	50395 (97.1)	<0.001	8707 (16.8)	43211 (83.2)	<0.001
Adequate	100942 (66.0)	3381 (3.3)	97561 (96.7)		16208 (16.1)	84734 (83.9)	
Sitting time							
Excess	84608 (55.3)	2661 (3.1)	81947 (96.9)	0.120	16387 (19.4)	68221 (80.6)	<0.001
Adequate	68252 (44.7)	2243 (3.3)	66009 (96.7)		8528 (12.5)	59724 (87.5)	
Cigarette consumption^*^							
Yes	7763 (5.0)	429 (5.5)	7334 (94.5)	<0.001	2212 (28.5)	5551 (7.5)	<0.001
No	19495 (12.8)	869 (4.5)	18626 (95.5)		5007 (25.7)	14488 (74.3)	
Alcohol consumption^†^							
Yes	34705 (22.7)	1095 (3.2)	33610 (96.8)	<0.001	8316 (24.0)	26389 (76.0)	<0.001
No	46414 (30.4)	1728 (3.7)	44686 (96.3)		9158 (19.7)	37256 (80.3)	
Drug use^‡^							
Yes	6239 (4.1)	315 (5.0)	5924 (95.0)	0.310	1765 (28.3)	4474 (71.7)	0.320
No	10005 (6.5)	470 (4.7)	9535 (95.3)		2759 (27.6)	7246 (72.4)	
School attendance							
Inadequate	24318 (15.9)	1033 (4.2)	23285 (95.8)	<0.001	5033 (20.7)	19285 (79.3)	<0.001
Adequate	128542 (84.1)	3871 (3.0)	124671 (97.0)		19882 (15.5)	108660 (84.5)	

*Cigarette consumption [(n=27258/missing=125602 (82.2)];

†Alcohol consumption [n=81119/missing=71741 (46.9)];

‡Drug use [n=16244/missing=136616 (89.4)].

p-value of the chi-square test.

Table 3 shows the results of the regression analyses for the association between lifestyle factors and mental health indicators. There was no multicollinearity between the model variables. According to the values expressed, infrequent healthy eating, being inactive or insufficiently active, screen time, smoking, and inadequate school attendance were associated with higher odds of mental health problems when using the number of close friends. On the other hand, frequent unhealthy eating and drinking was associated with lower odds for the same risk parameter. These values remained significant after adjusting for sex, age, and region, except for the screen time factor. When using self-rated mental health, the factors associated with higher odds for a negative rating were infrequent healthy eating, frequent unhealthy eating, being inactive or insufficiently active, excessive screen time and sitting, smoking, alcohol and drug consumption, and inadequate school attendance. All values remained significant after adjustment.

**Table 3:** Logistic regression showing the association of lifestyle factors with mental health indicators.

Factors	Close friends (no friends – risk)		Self-rated mental health (negative)	
	OR (95%CI)	Adjusted OR (95%CI)	OR (95%CI)	Adjusted OR (95%CI)
Healthy eating (ref. = Frequent)				
Infrequent	1.26 (1.18–1.36)^*^	1.22 (1.14–1.31)^*^	1.37 (1.33–1.42)^*^	1.29 (1.24–1.33)^*^
Unhealthy eating (ref. = Infrequent)				
Frequent	0.84 (0.79–0.90)^*^	0.86 (0.81–0.92)^*^	1.61 (1.56–1.65)^*^	1.39 (1.35–1.43)^*^
Physical activity level (ref. = Active)				
Inactive	1.85 (1.68–2.04)^*^	1.82 (1.65–2.01)^*^	1.83 (1.75–1.92)^*^	1.12 (1.07–1.18)^*^
Insufficiently active	1.16 (1.10–1.25)^*^	1.22 (1.14–1.31)^*^	1.22 (1.18–1.26)^*^	1.09 (1.05–1.12)^*^
Screen time (ref. = Adequate)				
Excess	0.87 (0.82–0.93)^*^	0.90 (0.85–0.96)^†^	1.05 (1.02–1.08)^*^	1.10 (1.07–1.14)^*^
Sitting time (ref. = Adequate)				
Excess	0.96 (0.90–1.01)	0.96 (0.91–1.02)	1.68 (1.64–1.73)^*^	1.68 (1.63–1.73)^*^
Cigarette consumption (ref. = No)				
Yes	1.25 (1.11–1.41)^*^	1.24 (1.10–1.40)^*^	1.15 (1.09–1.22)^*^	1.26 (1.19–1.34)^*^
Alcohol consumption (ref. = No)				
Yes	0.84 (0.78–0.91)^*^	0.81 (0.75–0.87)^*^	1.28 (1.24–1.33)^*^	1.33 (1.28–1.37)^*^
Drug use (ref. = No)				
Yes	1.08 (0.93–1.25)	1.09 (0.94–1.26)	1.04 (0.97–1.11)	1.13 (1.05–1.22)^*^
School attendance (ref. = Adequate)				
Inadequate	1.43 (1.33–1.53)^*^	1.31 (1.22–1.40)^*^	1.43 (1.38–1.48)^*^	1.53 (1.48–1.59)^*^

Logistic regression adjusted for sex, age, and region. Ref.: reference.

^*^p<0.001;

†p<0.05.

OR: odds ratio; CI: confidence interval.

Table 4 indicates the OR according to the number of recommendations fulfilled using those who did not achieve any of the recommendations as reference. Data that did not belong to any of the options were excluded. The results revealed that achieving any recommendation was associated with lower odds for negative mental health when compared to those who did not meet any of them (OR 0.89; 95%CI 0.85–0.92). The same was seen for those who accomplished two or three recommendations (OR 0.67; 95%CI 0.64–0.69 and OR 0.67; 95%CI 0.64–0.71, respectively). Attaining all movement recommendations plus frequent healthy eating and infrequent unhealthy eating reduced the odds of a negative self-rated mental health (OR 0.52; 95%CI 0.47–0.58). There was no statistical significance when using the number of close friends as a mental health indicator.

**Table 4:** Logistic regression showing the association of a set of healthy lifestyle factors with mental health indicators compared to those that did not meet any of the recommendations.

Factors	Close friends (no friends – risk)		Self-rated mental health (negative)	
	OR (95%CI)	Adjusted OR (95%CI)	OR (95%CI)	Adjusted OR (95%CI)
Compliance with one of the movement recommendations^1^(n=56207)	1.09 (1.01–1.19)	1.06 (0.97–1.15)	0.84 (0.82–0.88)^*^	0.89 (0.85–0.92)^*^
Compliance with two of the movement recommendations^1^(n=3532)	1.11 (1.02-1.20)	1.07 (0.98–1.16)	0.63 (0.60–0.65)^*^	0.67 (0.64–0.69)^*^
Compliance with all movement recommendations^1^(n=16406)	0.92 (0.82–1.04)	0.87 (0.77–0.98)	0.52 (0.49–0.55)^*^	0.67 (0.64–0.71)^*^
Active, adequate screen time, adequate sitting time, frequent healthy eating, and infrequent unhealthy eating^2^(n=4991)	0.88 (0.74–1.06)	0.88 (0.73–1.07)	0.36 (0.33–0.40)^*^	0.52 (0.47–0.58)^*^

^1^ Reference: Not achieving any of the recommendations for physical activity, screen time, and sitting time (n=26715). All other quantities of compliance with the recommendation were excluded of the analysis;

^2^ Reference: No movement recommendations, infrequent healthy eating, and frequent unhealthy eating; Movement recommendations: be active, adequate screen time, and adequate sitting time; Logistic regression adjusted for sex, age, and region.

*p<0.001.

OR: odds ratio; CI: confidence interval.

## DISCUSSION

The objective of the present study was to evaluate the association between modifiable lifestyle behaviors and mental health indicators in Brazilian adolescents. The results showed that eating habits, amount of PA, sedentary behaviors, consumption of psychoactive substances (cigarettes, alcohol, and drugs), and school attendance are associated with mental health. Furthermore, the results indicated that meeting current lifestyle recommendations is connected with lower chances of displaying mental health risk indicators.

In this study, the number of close friends was considered an indicator of mental health since it is widely recognized that close and positive relationships during adolescence play a crucial role in mental health and brain activation. Such bonds promote more effective modulation of affective emotions and better emotional control, contributing to a more rewarding sensation in social life.^16^ Our results showed that the level of PA and school attendance are behaviors associated with this indicator. A significant amount of physical activity during adolescence is more likely to be achieved through sports and games. Typically, these activities take place in settings that facilitate interaction among teenagers, strengthening friendships and enhancing social interaction during this phase.^17,18^ Following the same line of reasoning, we can justify the association between inadequate school attendance and a higher likelihood of not having close friends. School, besides providing education, is an environment conducive to teenagers establishing social contacts, which can facilitate the formation of close friendships.^19^ Furthermore, schools have physical education classes that also favor the affective and social development of adolescents.^18^ Missing classes can be an indication that the teenager is not happy with the school environment, and not having close friends can be a significant reason for not going to school.

The results also showed that alcohol consumption was associated with lower chances of not having friends. This correlation can be attributed to alcohol often being associated with social events and celebrations. Additionally, having close friends who drink alcohol may increase the likelihood of alcohol consumption.^20,21^ These factors may justify the relationship between more friendships and alcohol consumption. However, despite alcohol consumption and having close friends showing positive associations, this behavior is also correlated with several other risk indicators for mental and physical health,^22^ especially in adolescence, generating more harm than good. In addition to the negative effects already reported in the literature,^22^ the present study demonstrated a negative linkage between self-assessment of mental health and alcohol consumption.

Several lifestyle behaviors we analyzed were connected with self-rated mental health, indicating a positive association between healthy habits and better self-rated mental health. These data are in agreement with the literature and show that a healthy lifestyle promotes better quality of life and well-being for adolescents.^23^

Multinational studies have demonstrated a relationship between various mental health indicators and eating habits, regardless of the country or region.^24,25,26^ Studies in low-, middle-, and high-income countries demonstrated a positive association of the consumption of soft drinks and fast food with suicide attempts.^24,25^ In another analysis, carried out in the Caribbean, a connection of low consumption of fruits and vegetables with the feeling of loneliness was indicated.^26^ Our data also showed greater chances for negative self-rated health with infrequent eating of healthy food, as well as with frequent eating of unhealthy food. Similar results were presented in other cross-sectional studies^24,25,26^ but due to a lack of longitudinal data, these results need to be interpreted with caution. However, based on these data, it can be argued that, in addition to the positive physiological consequences of a balanced, nutrient-rich diet along with a healthy eating pattern and restricting unhealthy foods, there is an association between dietary habits and mental health in adolescents. This may also reflect a more structured family environment and other healthy behaviors that affect mental health.

PA and sedentary behavior were also revealed as important correlates of mental health in adolescents. Our data showed that inactive adolescents were more likely to have worse mental health indicators. Those considered insufficiently active also had higher chances of displaying indicators of poor mental health, although of a smaller magnitude compared to inactive ones. These results corroborate the idea that a little PA, even if insufficient, is better than nothing.^27^ Several studies compared health outcomes only between “achieving the PA recommendation or not”; however, separating the inactive from the insufficiently active should be further explored in the literature.^27^ We believe that changes in PA behavior occur in stages, and that there is some value in the change from inactive to insufficiently active. Reporting only the benefits of achieving PA recommendations may hide the benefits of those who add some PA a week, compared to those who do none.^27^

The use of psychoactive substances (cigarettes, alcohol, and drugs) also showed a greater risk of worse self-rated mental health. This behavior probably symbolizes the main risk to mental health.^22,28^ Several studies demonstrated the harmful effects of these substances on the brain, affecting health and development in various ways.^22,28^

More and more studies considered inadequate school attendance as a potential health risk factor. Systematic reviews already investigated the effects of this behavior on mental health, and revealed associations with different mental health indicators, such as anxiety disorders, depression, and social phobia.^29^ The present study evidenced that inadequate school attendance was associated with mental health indicators. Furthermore, it is important to consider that this situation can be influenced by several contextual factors, such as perceived violence in the school region or in the neighborhood where the adolescents live. Exposure to violence can create a hostile school environment, contributing to increased absenteeism among students. Problems related to school transport, such as difficulties in accessing safe and reliable public transport, can also influence adolescents’ school attendance, leading to frequent absences. The family’s financial situation can also be a risk factor to be considered. As advised by other studies,^29^ we emphasize that school employees and doctors pay attention to adolescents’ school presence, as this is a behavior that can serve as a sign of risk to mental health.

Since many adolescents who did not meet PA recommendations had excessive screen and sitting time, we investigated the chances of mental health indicators according to compliance with one or more movement recommendations (being active, adequate screen and sitting time). The results showed a progressive decrease in negative self-rated health as the number of recommendations achieved increased. These data indicate that accumulating healthy lifestyle behaviors can reduce the chances for mental health problems and attaining at least one recommendation would already provide a benefit compared to those who do not achieve any of them. Furthermore, combining appropriate movement-related behaviors with healthy eating behaviors can provide additional mental health benefits.

In accordance with the ecological model for health promotion, this study focused on exploring behaviors considered intrapersonal factors and not on interpersonal or community factors, such as parental support or leisure conditions that depend on public policies.^30^ These intrapersonal factors are more dependent on the individual and are characterized by attitude and behavior.^30^ Nevertheless, personal choices can be influenced by environmental factors like access to open space or facilities for PA and affordable healthy food choices. The data, however, indicates that small behavioral changes can already provide benefits for mental health or lower the risk of displaying indicators of negative mental health. For example, moving from the category inactive to insufficiently active would already decrease the risk for worse mental health. At the same time, complying with any of the movement recommendations would generate a risk reduction compared to those who did not achieve any recommendations.

The present study has limitations that must be taken into account:

1. The cross-sectional nature does not allow for interpretations of causality between behaviors and mental health indicators but it does allow to determine associations;
2. The use of questionnaires has a risk for recall bias, which can lead to incorrect interpretations of questions by adolescents; however, the research was based on large-scale school health surveys that have been conducted throughout the world^7,13^ such as the Global School-Based Student Health Survey, the Health Behavior in School-aged Children, and the Youth Risk Behavior Surveillance System;
3. The generalization of data must be viewed with caution due to sample loss according to the exclusion criteria, despite the fact that the sample population consists of Brazilian adolescents from all regions of the country; and
4. The data were obtained from adolescents enrolled in schools, excluding those not enrolled in the Brazilian education network, even though the PeNSE was applied in indigenous and remote regions of Brazil. Despite these limitations, the size of the research and its national scope allows for viable insights into the association between lifestyle behaviors and mental health in Brazilian adolescents.^7,8^

We conclude that lifestyle behaviors are associated with mental health indicators and that the accumulation of healthy behaviors can progressively reduce the chances of presenting a negative self-rated mental health indicator. Furthermore, even if adolescents do not meet the PA recommendations, doing some PA may be better than none. Public policies, therefore, must outline strategies to encourage changes in lifestyle behavior among adolescents and prevent adolescents from engaging in risky behaviors.

## Data Availability

The database that originated the article is available in a open repositor: National Survey of School Health (PeNSE, *Pesquisa Nacional de Saúde do Escolar*). The PeNSE is maintained by the Brazilian Institute of Geography and Statistics (IBGE, *Instituto Brasileiro de Geografia e Estatística*).
